# 509. Burn Injury as a Chronic Health Condition: Survivor-Reported Symptoms and Demographic Disparities

**DOI:** 10.1093/jbcr/irag033.168

**Published:** 2026-04-06

**Authors:** Jill L Sproul, Rachel Kudlak, Daniel W Chacon, Amy L Acton, Sandra J Cramolini, Liliana Palacios, Sara E Byrne

**Affiliations:** Phoenix Society for Burn Survivors, San Jose, CA; Phoenix Society for Burn Survivors, Charleston, SC; Phoenix Society for Burn Survivors, Fresno, CA; Phoenix Society for Burn Survivors, Kentwood, MI; Phoenix Society for Burn Survivors, Knoxville, TN; Phoenix Society for Burn Survivors, Oakland, CA; Phoenix Society for Burn Survivors, Grand Rapids, MI

## Abstract

**Introduction:**

Burn injuries are often treated as acute events, yet many survivors experience physical, psychological, and social challenges that persist for years or decades. Framing burn injury as a chronic health condition can better align services with long-term needs and equity. We characterized survivor-reported symptoms, concerns, and community priorities by time since injury, age at injury, burn severity, and demographics to delineate the enduring, systemic nature of burn recovery.

**Methods:**

A cross-sectional, English language, self-report survey was completed by burn survivors (n = 421). Variables included time since injury, total body surface area (TBSA), age at injury, visible scarring, health experiences in the past 12 months, current symptoms and conditions, survivor concerns, and satisfaction with support quality. Demographic variables (language, education, race/ethnicity, household income) were included to examine equity considerations. Analyses were descriptive (frequencies, percentages, means, and standard deviations). Because not all respondents answered every item, the number of responses varies by question and is reported for each result.

**Results:**

Nearly all respondents reported visible scarring (99.3%, n = 407). Time since injury: 52.5% (n = 216) were > 10 years post-injury and 26.0% (n = 107) were 1–4 years out; 51.8% (n = 210) were injured under the age of 18. Burn size varied widely: over one-third <10% TBSA and more than a quarter ≥30% TBSA. In the past 12 months, common problems were dermatologic 75.5% (n = 265), contractures 70.1% (n = 246), temperature regulation 69.0% (n = 242), mental health 65.0% (n = 229), pain 58.1% (n = 204), and insomnia 53.8% (n = 189). Ongoing conditions mirrored these domains; issues related to skin 69.5%, sleep/fatigue 59.9%, musculoskeletal 53.1%, neurologic/psychiatric 51.7%, metabolic 50.6%). Top needs were coping tools, survivor connection, and advocacy 95% (n = 343). Support quality ratings varied (40% excellent; 17% low). Demographics showed a largely White (68%), English-speaking (92%) sample with substantial financial insecurity (22% < $25 k; 27% $25–$49 k).

**Conclusions:**

Findings reinforce burn injury as a chronic condition: survivors report persistent multidomain symptoms, psychosocial concerns, and community needs well beyond the acute phase. Long-term burden varies by injury severity, time since injury, and age at injury, and demographic differences indicate inequities.

**Applicability of Research to Practice:**

Findings support a chronic care approach to burn recovery: proactive screening, survivor centered mental health resources, and equity informed programming. Aligning services with survivor priorities (connection, coping tools, advocacy) can raise engagement and outcomes, while addressing disparities in income, education, and race improves access and fit.

**Funding for the study:**

N/A.

